# Preschool-based mother-child emotional preparation program improves emotional connection, behavior regulation in the home and classroom: a randomized controlled trial

**DOI:** 10.3389/frcha.2023.1232515

**Published:** 2023-10-20

**Authors:** Martha G. Welch, Robert J. Ludwig, Amie A. Hane, Judy Austin, Elizabeth S. Markowitz, Marc E. Jaffe, Michael M. Myers

**Affiliations:** ^1^ Department of Pediatrics, Columbia University Irving Medical Center, New York, NY, United States; ^2^ Department of Psychiatry, Columbia University Irving Medical Center, New York, NY, United States; ^3^ Department of Psychology, Williams College, Williamstown, MA, United States; ^4^ Mailman School of Public Health, Columbia University, New York, NY, United States; ^5^ Children's Learning Centers of Fairfield County, Stamford, CT, United States

**Keywords:** emotional dysregulation, classroom behavior, relational health, socioemotional behavior, family intervention, ASQ questionnaire, autism treatment evaluation checklist, Welch Emotional Connection Screen

## Abstract

**Introduction:**

Based on the theory that increasing emotional connection and reducing emotional stress between mother and child at home will reduce dysregulated behavior in the classroom, we tested a novel family-based school intervention aimed at facilitating mother-child emotional connection. This question has gained great importance following the COVID-19 pandemic, as child mental health has been declared a national emergency.

**Methods:**

Subjects were randomized into two groups; one (Control: *n* = 32) receiving the standard curriculum in a large community-based preschool education program, and another (MCEP) receiving the standard curriculum plus the Mother Child Emotional Preparation Program (MCEP: *n* = 30). Two to eight MCEP mother-child pairs participated in eight elective weekly 2-h group sessions over a 16-week period. During the 2-h sessions, the pairs were engaged in face to face calming sessions. At 6 months post-enrollment, we assessed mother-child emotional connection on the Welch Emotional Connection Screen (WECS). In addition, mothers and teachers completed validated questionnaires and instruments.

**Results:**

We found that the percentage of MCEP mother-child pairs who became emotionally connected at 6 months was five-fold higher than Control pairs (47.4%, vs. 8.3% *p* = 0.004, effect size = 0.44). Also at six months, MCEP children had fewer behavioral symptoms (*p* = 0.024)) (effect size >0.5); fewer autism symptoms (*p* = 0.048) (effect size = 0.53); fewer emotional symptoms (*p* = 0.01) (effect size >0.76); better personal, social skills (*p* = 0.045) (effect size = 0.51); better executive function (*p* = 0.032) (effect size = 0.59). Importantly, teachers reported MCEP children showed more improved behavior in the classroom, compared to controls.

**Discussion:**

This trial was retrospectively registered in the clinicaltrial.gov registry (NCT02970565) on April 9, 2019.

## Introduction

Antisocial behavior in preschool-aged children has been increasing for decades in the US (1, 2). Preschoolers now have the highest rates of school expulsion of all age groups. Such adverse behavior in childhood often leads to lifelong social exclusion and considerable personal distress into adulthood (3) (4), and imposes high public and private expenditure for treatments (5–7).

During the conduct of this study, the problem worsened as the result of the COVID-19 pandemic. The situation has left teachers increasingly burned out—A recent survey of over 4,500 preK-12 teachers at nationwide public, private and charter schools (8) asked, How has your teaching changed during the 2021–2022 year? Among other categories, respondents reported

• Overall workload increased 81%;
• Spending more time addressing students’ mental health increased 80%;
• Classroom interruptions during instruction increased 45%.

Over the last 10 years, numerous Cochrane reviews and meta-analyses have examined the efficacy and effectiveness of various intervention programs designed to tackle the rising problem of emotional, behavioral, and developmental disorders in preschool-aged children. Interventions include psychosocial interventions for ADHD (9), psychological interventions targeting behavioral inhibition and anxiety (10), cognitive behavioral therapy (CBT) (11), school-based interventions to prevent anxiety and depression in young children (12), Incredible Years Teacher Classroom Management (IYTCM) for adverse socioemotional behavior (13) and Parent Management Training (PMT) for behavior problems (14).

There are numerous interventions for preschool children that treat parents and children together. Parent-Child Interaction Therapy (PCIT) is a well-established treatment for behavioral, hyperactivity and oppositional-defiant problems in children, with more than 40 years of research behind it (15). A variation of PCIT, PCIT-ED, focuses on emotion development (16). Cool Little Kids, focuses on anxiety (17) and Child-parent psychotherapy (CPP) is a well- studied psychodynamic treatment that targets the parent's and the child's experience of their relationship as the most

Some preschool-based interventions include families. However, the focus of these interventions is on improving the individual child's development. For instance, Ray et al. (18) developed the Increased Health and Wellbeing in Preschools (DAGIS) intervention to enhance preschoolers’ self-regulatory abilities and energy balance-related behaviors through a program involving educators and the children's families. However, results demonstrated no improvement when accounting for parental education level. Kochanska (19) found that in a longitudinal study of parent-preschool-aged child pairs, high “mutual responsive orientation,” corresponded to mothers using less power and children internalizing more maternal values and rules. A recent meta-analysis showed evidence that parenting programs integrated into early childhood education may have an effect on children's behavioral outcomes (20). While these studies involved family members, their outcome measures concentrated on the child or on the mother alone, not on the dyad.

The systematic reviews listed above point out that some interventions for preschool-aged children have shown both efficacy and effectiveness. However, nearly all reviews conclude that current interventions show limited or inconclusive effects on overall adverse classroom behavior. Another common conclusion is that due to small effect size and due to the length and cost of treatment programs, current treatment models are not suitable in their present form for wide scaling that would meet the emergent needs (21, 22). The shortage of effective behavioral interventions has left preschool educators struggling to find alternative solutions.

To address the problem, we developed a novel school-based program—Mother-child Emotional Preparation (MCEP) in partnership with the Children's Learning Centers of Fairfield County (CLC), a leading community-based pre- school education program serving ∼1,000 families annually in Stamford, CT. The challenge was to develop a scalable, low-cost group treatment model that would avoid placing significant financial, administrative or teaching burden on the Center's already stretched resources. The goal was to reduce classroom behavioral disturbances such that the teachers could deliver their curriculum with fewer disruptions. MCEP is a preventative group family intervention that is designed to help parents prepare the child emotionally for the pre-school educational experience. MCEP is based on calming cycle theory (23). The theory predicts that repeated mother-child calming sessions can reduce child adverse behavior at home and in school.

MCEP is novel in its engagement of both mother and child in a full range of emotional expression during mutual sensory calming sessions. Our hypothesis is that empathy is evoked when the mother (or other person) expresses the full range of their deep feelings. When a child responds to the mother's expression of emotion and shows signs of empathy to the mother, a temporary break in connection begins to repair. Repair needs to be mutual and on-going to be effective for both members of the pair. Both the mother and child learn that repair can be achieved through emotional communication. The connected child is thereafter able to connect and co-regulate with teachers and others once the pattern is established with the mother and family members. CLC leadership has reported that the children who were in the MCEP program changed the classroom environment with their positive affect and better regulated behavior so much so that teachers were freer to address problems such as learning difficulties of the non- symptomatic children for whom teachers did not have the band-width to address prior to MCEP.

We found that MCEP improved mother-child emotional connection as assessed by the WECS and significantly improved child behavior at home and in school. We discuss the implications of our findings for preschool education, along with strengths and limitations of the study.

## Methods

### Trial design

The MCEP study was a parallel-group, single blind RCT that was approved by the Columbia University IRB (AAAT0109). Subjects for this study were a non-probability convenience sample of mainly low socioeconomic status families. The method is presented as per the CONSORT guidelines (24). The trial was prospectively registered in the clinicaltrial.gov registry (NCT02970565).

### Participants and setting

The study was conducted at Children’s Learning Centers of Fairfield County in Stamford, CT, a community-based preschool education center. CLC has eight sites and three programs: School Readiness, Child Development and Head Start (25). Families must meet various criteria to be eligible for enrollment in one of CLC's programs (Head Start & Early Head Start, Child Development and School Readiness). According to CLC's Parent Manual (25), programs vary by funding and eligibility requirements. Head Start is a free federal program that operates under a standardized curriculum with eligibility based on poverty. In the Child Development program, parents must work 30 h per week to be eligible for specific Connecticut-funded programs (25).

### Recruitment

We recruited families continuously from all CLC programs. Study staff recruited at orientations, teacher/parent meetings and distributed flyers during child drop-off/pick-up. Teachers signed a consent form to collect teacher- report data. CLC staff discussed the study with parents and encouraged teacher referrals. The primary recruitment method was recruiting mothers in-person during child drop-off/pick-up.

### Eligibility

Children were eligible for the study if they were between the age of two and four- and one-half years of age at the recruitment date. In addition, the child had to be a singleton without any genetic or congenital disorder or motor disability. Mothers had to be at least 18 years old, able to speak, read and write in English or Spanish; and be living with her child full-time. Exclusion criteria for mothers included: severe mental illness or any other medical conditions preventing play activities; involvement with the Department of Children and Families; struggling with drug or alcohol abuse; pregnancy (second trimester) that could interfere with the lap-based procedures (see below); or unable to commit to the study schedule. Demographics for each group at each time point are presented in Supplementary Figure S1.

### Consent procedures

After verbal consent, mothers completed Study Eligibility and Demographics Forms, and the CLC Release information sharing in person or by phone, with the CLC Release signed at the first in-person contact. Mothers signed a consent form at the time of the baseline assessment and then, if allocated to the intervention arm, were assigned to a MCEP group.

### Randomization

**Figure 1:**
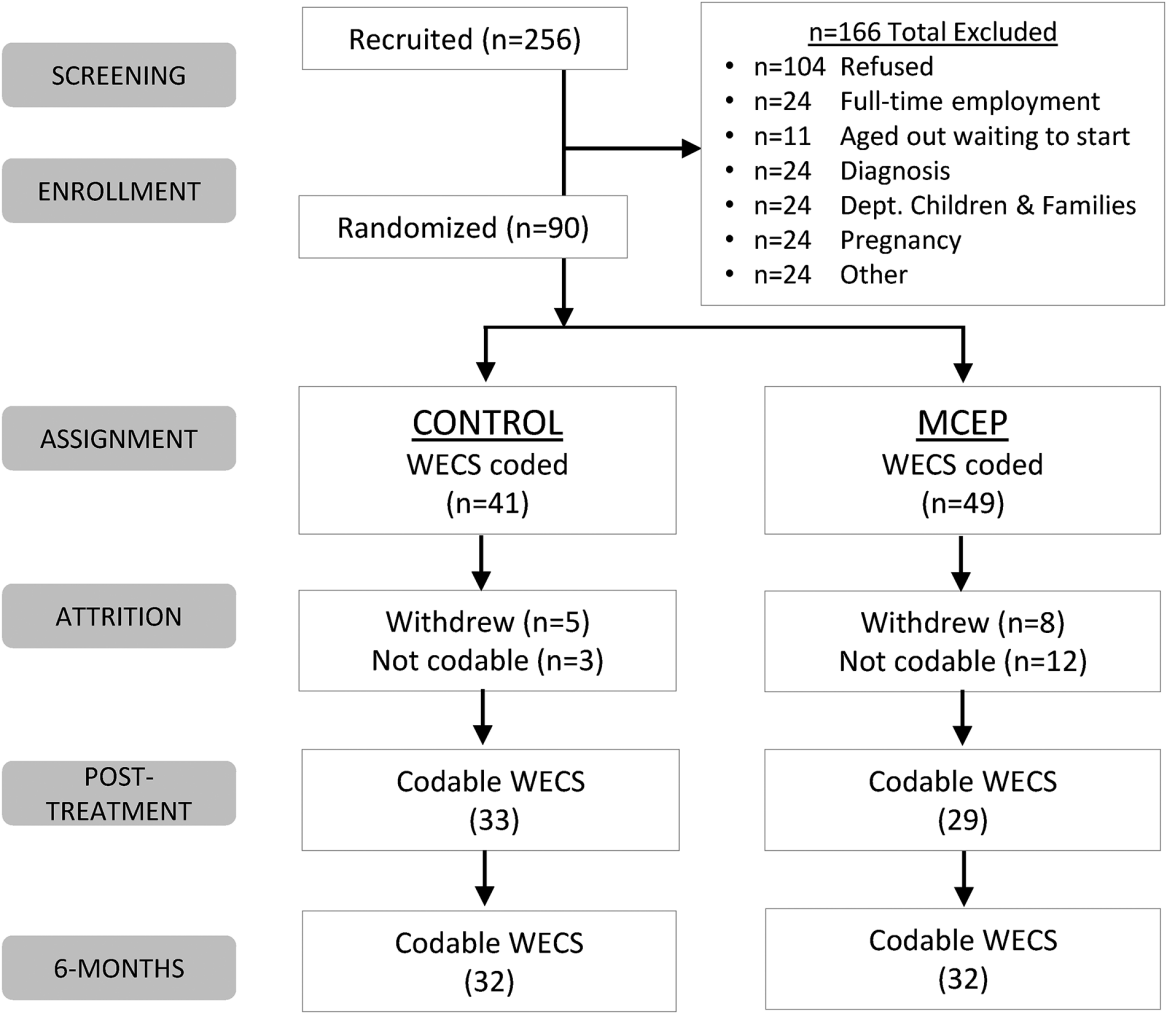
Consort chart. WECS, welch emotional connection screen; MCEP, mother-child emotional preparation; *n* = sample size.

Participants arrived 30 min before the first MCEP session for group assignment. Prior to first subject enrollment, a computer-generated block randomization sequence ensured balanced assignment. Based on the group's size as estimated prior to the first session, cards denoting MCEP or Control were placed in envelopes. Upon arrival, we handed the mother a sealed envelope. If control, we informed the mother she was randomized to the CLC Standard Curriculum and that she may leave. If intervention, we asked mothers and children to stay for the first MCEP group session. See Consort flow Chart (Figure 1) for final group assignment numbers. Note: In Figure 1, the number of subjects shown at enrollment (baseline), and at the first and second follow-up (approximately 2 and 6 months) reflect the number of subjects with scorable videos. The numbers of subjects with questionnaire data were, in some cases, different from the number with videos.

### Control group

Control group children participated in the CLC standard curriculum with no additional procedures. Classroom activities varied by age and ability, and classroom structure varied by program. All CLC programs used the Connecticut Early Learning and Development Standards and the Connecticut Preschool and Assessment Framework combined with the Creative Curriculum (25). CLC's Head Start Program uses the Creative Curriculum, in addition to the Head Start Child Development and Early Learning Framework. Child Development and School Readiness programs used Connecticut Data Observation and Tracking System, and CLC's Head Start and Early Head Start sites use the Teaching Strategies Gold system to track student progress (25). CLC teaches emotional literacy through the Yale RULER curriculum at all sites.

### Intervention methods

Mother-child dyads randomized to the intervention arm received facilitated MCEP group calming sessions. In this study, two specially trained Nurture Specialists, both licensed clinical social workers, facilitated the parent-child intervention and provided emotional support during sessions. Each mother-child dyad participated in two to eight 2-h group sessions over 16 weeks, which we held in a classroom or small meeting room.

As described in prior literature (26, 27), calming sessions consisted of a child sitting on the mother's lap and cycling through a range of verbal and non-verbal emotional expressions. A key goal of the session was to assure the dyad maintained close physical and sensory contact throughout the session.

This communication typically dealt with current or past upsets or other previously unprocessed feelings. A successful calming session included four phases. In the first phase, DISCOMFORT AND DISTRESS, the mother and child displayed and expressed separate distress for routine topics. Children sometimes rejected their mother's request to sit and talk by wriggling away, becoming restless and dysregulated, or by being unable to control their own emotional state.

The second phase, CONFLICTS AND UPSET, involved the mother and child expressing upset about the other's behavior. The child's *separate distress* often prompted the mother to feel her own disrupted connection. The Nurture Specialist helped the mother and child identify and express their feelings to one another. For example, Mom, tell your child: how you feel when he/she doesn't look at you; how you want to feel when you're together; how you feel when you are apart from them; How you feel when your child screams/hits/kicks. How you feel during a tantrum.

The child was prompted to tell mom: what makes you mad, sad or worried; how you feel when mom goes to work; how you feel when you can't buy candy; when you are worried about mom. Sometimes in this phase, the mother cried (e.g., feeling sadness discussing separation or joy at the story of the birth), which also led to the child orienting to her and to feel her emotional state. The child often responded to the mother's upset with tender behaviors and empathic communication. Mothers were encouraged to use comforting touch, genuine emotional expression, soothing, and eye contact to mutually resolve the upset and bring each other into a calm state.

Once the upset was fully processed, the dyad typically began to soften toward one another and reached mutual resolution. This signaled the beginning of the third phase, CONFLICT RESOLUTION. In this phase, Nurture Specialists used verbal prompts to help the mother elicit her deep emotion by tapping into her emotional memories, by way of asking to tell the child her birth story or other stories when she was the child's age. In response to the mother's emotional expression, the child will often orient to the mother's face with direct gaze, rapt attention and, often, loving touch. Once conflicts and upset were processed and the two were oriented to one another without rejection, they reciprocated by tenderly hugging and gazing at each other warmly and began to discuss good as well as upsetting behavior (e.g., child noncompliance or maternal inattention or separation), and talked about plans to avoid upsets.

The final stage of the calming session, MUTUAL CALM, was characterized by cuddly closeness, with mother and child breathing quietly, maintaining a deep mutual gaze and warm, open verbal and non-verbal communication, with observable relaxation and reciprocal pleasure in each other's embrace. Following the first session, mothers made a brief report of their home calming sessions (e.g., handling of tantrums and child noncompliance). Following each session, the Nurture Specialists encouraged mothers to continue preventative and reparative calming sessions on a regular basis at home, especially during conflict, tantrums or signs of upset.

### Assessment tools

#### Welch emotional connection screen (WECS)

We measured mother-child emotional connection using the Welch Emotional Connection Screen (WECS) assessment tool (28). WECS concurrent validity was demonstrated using indicators of emotional connection coded in observational software by independent teams of coders. The WECS construct validity was established by showing pairs rated emotionally connected had healthier autonomic responding in the still-face paradigm (SFP) and more approach-seeking behavior with mother during the recovery phase of the SFP (28). WECS predictive validity was shown by demonstrating pairs rated emotionally connected at age 6 months had fewer behavior problems at age 3, as reported by mothers (29).

We trained a team of six coders blinded to all other study data on a remote platform. Coders first achieved reliability with scores of the WECS creators on a training set of mother and preschool-aged child videos derived from other samples, with each coder required to achieve an intra-class correlation for each dimension of the WECS of at least 0.85. Thereafter, the team advanced to coding 13 cases from the current CLC trial sample. We established further reliability by having coders cross-code one another's cases. Coders achieved reliability with one another individually with scores no lower than 0.85. The average intra-class correlation coefficients for each dimension of the WECS pooled across coders were: Attraction = 0.93, Vocal = 0.93, Facial = 0.95, Sensitivity/Reciprocity = 0.94. Each study pair had repeated WECS observations. The baseline and post-treatment WECS scores were coded by different coders, such that no blinded coder coded the same dyad more than once.

The WECS composite emotional connection score is made up of four behavioral modes of expression, as defined by Hane et al. (28). They are:

1. Mutual Attraction (shared gaze, mutually seeking physical closeness and proximity)
2. Mutual Vocal Communication (warmth in vocal tone and amount and content of vocal behavior of mother; consistent and warm vocal responsiveness from child; connection through voice);
3. Mutual Facial Communication (expressions of positivity, laughter, joy and and sustained eye gaze);
4. Mutual Sensitivity and Reciprocity (well-timed reciprocal social sensitivity to each other's expressed emotions).

The WECS is scored on a nine-point Likert scale, starting at 1.00. The lowest rating corresponds to the least emotionally connected. Scores increase by .25 increments up to a total of 3.00, which corresponds to the most emotionally connected rating. The four separate mode scores are totaled to give a WECS total score. A score equal to or greater than 9.00 was the threshold for determining whether a study pair were emotionally connected. In a few cases, it was not possible to score one of the modes of expression. In these cases, we totaled the other three scores, divided by three and multiplied by four to obtain an estimate of the total score.

#### The Welch orienting lapcheck

**Figure 2:**
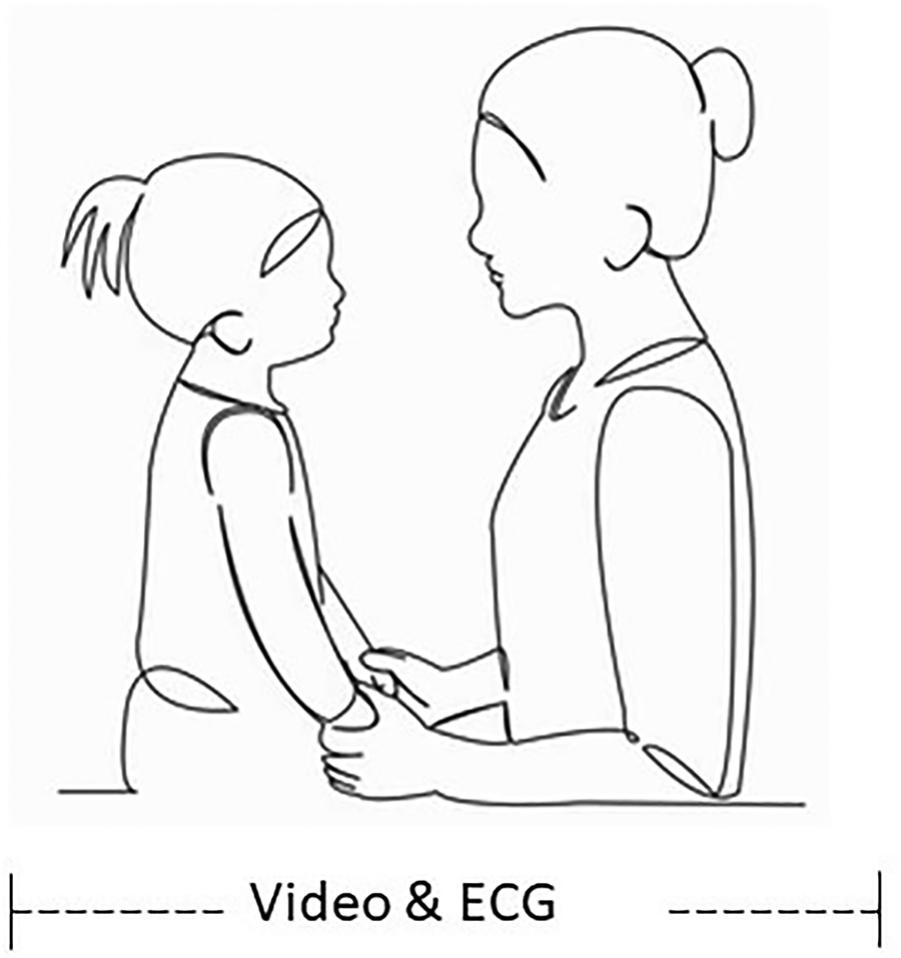
Schematic showing face to face orientation during Welch Orienting Lapcheck. The face to face orientation and physical contact is necessary to trigger the autonomic socioemotional reflex (ASR). The behaviors that result from the autonomic reflex are measured using the Welch emotional connection screen (WECS) and determine whether the autonomic “states” of the pair are “connected” or “disconnected”.

We used the Welch Orienting Lapcheck (Figure 2) to test the pair's emotional reaction to one another. This test is designed to capture the behavior and physiology associated with the mother and infant/child autonomic socioemotional reflex (ASR) (30) which is triggered when the two encounter one another physically and emotionally face to face. The test begins when the mother seats her infant on her lap or asks her child to sit on her lap. The mother was instructed to interact normally with her child for 2–5 min. The test is video-recorded and mother and infant/child are wired for electrocardiograms (ECG). Behaviors were coded with the Welch Emotional Connection Screen (WECS) (28).

#### Center for epidemiologic studies depression scale (CES-D)

We measured mothers’ depression on the CES-D. The CES-D is a 20-question self-report form that asks the individual to rate whether he or she experienced a given emotion (e.g., “I felt fearful”) “rarely or none of the time”, “some or a little of the time”, “occasionally or a moderate amount of time” or “most or all of the time” (31). Mothers completed the CES-D at the baseline, post-treatment and 6-month time points.

#### Survey of well-being of young children (SWYC)

The SWYC, a short, simple questionnaire completed by the parent of a child under 5 years, captures the parent's assessment of children's motor, language, social and cognitive development, as well as emotional/behavioral functioning, and any familial risk factors (32). It covers three domains of functioning: Behavior and Emotion, Family Risk Factors, and Development. Mothers completed the appropriate survey according to the child's age at each time-point.

#### Autism treatment evaluation checklist (ATEC)

The ATEC, developed by Rimland and Edelson (33) at the Autism Research Institute, was completed by mothers in this study. It consists of 4 subtests: I. Speech/Language Communication (14 items); II. Sociability (20 items); III. Sensory/Cognitive Awareness (18 items); and IV. Health/Physical/Behavior (25 items). We present results using the total score across these subtests.

#### Strength and difficulties questionnaire (SDQ)

The SDQ is a brief behavioral parental report questionnaire for children 3–16 years old (34). The version used in this study included 25 items divided between five scales: emotional symptoms, conduct problems, hyperactivity/inattention, peer relationship problems, prosocial behavior.

#### Ages and stages questionnaire (ASQ)

The ASQ was developed by Jane Squires and Diane Bricker (35, 36) and in this study was filled out by mothers. It consisted of six items covering five developmental areas: communication, gross motor, fine motor, problem solving, and personal-social.

#### Behavior rating inventory of executive function—preschool (Brief-P)

The Brief-P examines everyday behaviors associated with specific domains of executive functioning in children aged 2–5 years (37). It was completed by mothers in this study and includes 63 items in five non-overlapping scales forming a Global Executive Composite (GEC) and three overlapping summary indexes each with two scales based on theoretical and statistical considerations. The Inhibitory Self-Control Index (ISCI) is composed of the Inhibit and Emotional Control scales, the Flexibility Index (FI) is composed of the Shift and Emotional Control scales, and the Emergent Metacognition Index (EMI) is composed of the Working Memory and Plan/Organize scales.

### Blinding

Study staff (other than the NSs) outcome assessors, and coders were blinded to the randomization sequence. Mothers were not blinded.

### Statistical methods

The primary, pre-declared outcome for this RCT was the percent of dyads judged to be emotionally connected at the Post-treatment and 6-month time points. Emotional connection Yes/No codes were converted into 1/0 and analyses of covariance, controlling for baseline were used to test for intervention effects. Additional analyses compared groups’ emotional connection over time. Specifically, chi-squared analyses were used to test for group differences in the percentage of dyads that changed from not connected at baseline to connected at follow-up. Effect size calculations were based on either Cohen's-d or the Phi-coefficient.

We analyzed depressive symptoms (CES-D) using repeated measures ANOVA across the three data acquisition time-points (baseline, Post-Intervention and 6 month). We analyzed the effects of the intervention at the post- treatment and 6-month time points for the SWYC using analyses of covariance, controlling for baseline SWYC scores. We computed correlations (Pearson) to determine relationships between the WECS four factors and the WECS composite score and the SWYC (see Supplementary Table S2). These analyses were conducted for the overall group combining controls and intervention and then, for each group separately. Finally, we analyzed total scores or selected items from four additional questionnaires (ATEC, SDQ, ASQ, Brief-P) administered at the 6-month time- point using analyses of covariance, controlling for baseline scores.

## Results

### MCEP mother-child pairs were more emotionally connected at 6 months

The RCT primary outcome of this RCT was mother-child emotional connection, as measured on the Welch Emotional Connection Screen (WECS). The CONSORT flow diagram (Figure 1) shows the numbers of mother- child pairs from screening to enrollment between 2018 and 2020. We enrolled and group assigned at total of 90 pairs (41 control; 49 MCEP). Supplementary Table S1 provides demographic characteristics of pairs at three time-points.

**Figure 3:**
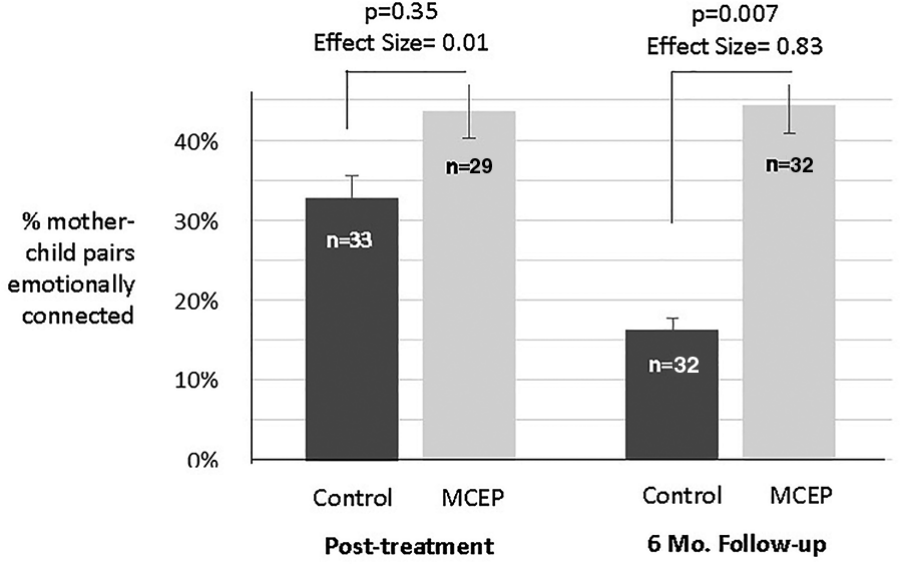
Mother-child emotional connection. Percent of pairs rated emotionally connected on the Welch emotional connection scale (WECS) at the end of the 8-weeks intervention and at 6 months follow-up. Note the % MCEP pairs emotionally connected remained approximately the same, while the % SC pairs emotionally connected actually dropped from 33% to 16%. MCEP, mother-child emotional preparation; *n* = sample size.

We hypothesized that a greater percentage of MCEP pairs (vs. Control pairs) would be emotionally connected post-treatment (end of intervention) and at the 6-month follow-up. Table 1 summarizes the effects of the intervention on emotional connection when assessed at post-tx (2-months) and 6-months. At Baseline and post- treatment there was no significant difference between groups. However, at the 6-months, a greater percentage of MCEP pairs (47%) were emotionally connected compared to controls (16%) (*p* − 0.007, effect size = 0.83) (see Figure 3).

**Table 1:** WECS scores at post-Tx and 6 months.

	Control	MCEP	
At Post-Tx	8.22 ± 0.22 (*n* = 33)	8.37 ± 0.23 (*n* = 29)	*F*(1,60) = 0.21, *p* = 0.65
	(Median = 8.50)	(Median = 8.67)	
At 6 months	7.43 ± 0.26 (*n* = 32)	8.61 ± 0.26 (*n* = 32)	*F*(1,62) = 10.56, *p* = 0.002, ES = 0.81
	(Median = 7.38)	(Median = 8.75)	

COVID had no effect on the treatment scores. Two-way ANOVA analyses on the interaction between intervention and COVID was not significant [*F*(1,60) = 1.00, *p* = 0.32]. MCEP, mother-child emotional preparation; *p* = Probability; ES, effect size; *n* = sample size.

A second set of analyses assessed how many pairs remained emotionally connected between post-intervention and six months. We found that at 6 months MCEP pairs remained connected, while Control pairs significantly decreased (47.4%, vs. 8.3% *p* = 0.004, effect size = 0.44) (see Supplementary Figure S1).

In addition to the above analyses based on the binary coding of WECS scores (EC+ or EC−, using a cut-off of 9 or better for EC+) we also ran ANOVAs using the total WECS scores across the four domains (attraction, vocal communication, facial communication, sensitivity). As for the binary coding, there was a highly significant effect of MCEP on WECS total scores at 6 months (Effect Size = 0.81). Below are the means (±SE) for WECS total scores at the Post-Tx and 6-month time points.

### MCEP mothers reported significant behavioral improvement at 6 months

#### SWYC preschool pediatric symptom checklist

**Figure 4:**
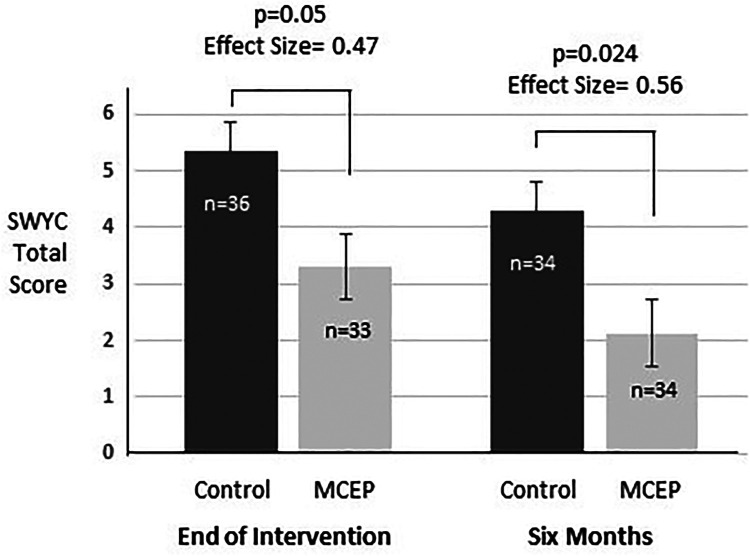
Comparison of SWYC scores. Total scores for the 18 item Survey of Well-Being of Young Children (SWYC) preschool pediatric symptom checklist at time-points 1 and 2. These analyses control for SWYC total scores at baseline (at time of enrolment). Baseline scores were included in these ANOVAs. MCEP, mother-child emotional preparation; *n* = sample size.

Figure 4 shows the values for the total number of symptoms reported by parents for four SWYC subscales (Externalizing, Internalizing, Attention Problems, and Parenting Challenges). At both the Post-Intervention and 6-month time points, MCEP significantly decreased the number of child behavioral symptoms (4.29 vs. 2.13, *p* = 0.024, with an effect size greater than 0.5.

Four domains of the WECS (mutual attraction, facial, vocal and sensitivity). Across all subjects, at the 6-month time-point, all WECS codes were negatively correlated with behavioral problems (i.e., better WECS scores were associated with fewer problems; only facial scores did not reach statistical significance). However, when broken down by group at 6 months, none of these correlations were significant in the control group whereas the total WECS, vocal and facial scores were significantly related to behavioral symptoms in the MCEP group (see Supplementary Table S2).

#### Four other parent-rated measures of behavior

**Figure 5:**
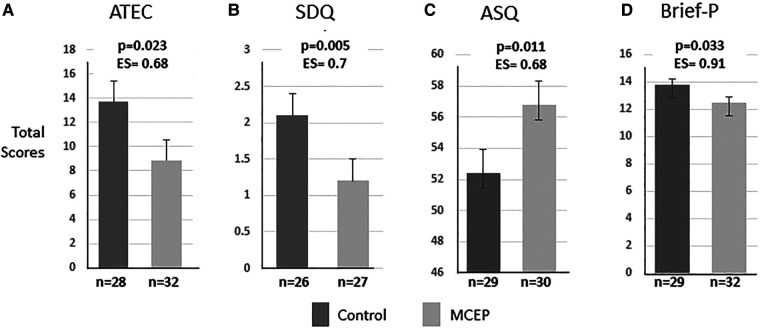
Child behavior outcomes at 6-month follow-up. Total scores on four widely used behavioral measures in preschool-aged children. The results for ATECT, SDQ, Brief-P were controlled for baseline. There was no baseline for ASQ. Note that MCEP scores were significantly better than controls on all four measures. (**A**) ATEC, autism treatment evaluation checklist—*fewer autism symptoms*. (**B**) SDQ, strength and difficulties questionnaire—*fewer emotional symptoms*. (**C**) ASQ, ages & stages questionnaire—*better personal, social skills*. (**D**) Brief-P, preschool version of the behavioral rating inventory of executive function—*better executive function*. Note that the large effect size (ES) indicates the intervention effect is large enough to be noticeable in the average size class. The analyses for each measure control for baseline (at the time of enrolment). MCEP, mother-child emotional preparation; *n* = sample size.

Six aspects of the WECS codes were analyzed; emotionally connected yes/no, total WECS score and scores for each of the four modes. Significant findings from analyses of four additional assessments are presented. There were no differences at baseline for ATEC, SDQ, and Brief-P. There was no baseline measure for ASQ. At six-month follow-up, these results show that MCEP children had fewer symptoms of autism (ATEC total score), fewer emotional problems (SDQ, emotional symptoms), better social interactions (ASQ, personal-social), and improved cognitive flexibility (Brief-P, Set shifting). (For complete statistics, see Supplementary Table S3). The effect sizes are robust, ranging from .68 to .91 for the four behavioral outcome measures at 6-month follow-up. This indicates strong sustained effects of the intervention (see Figure 5).

### Teachers reported MCEP children showed significant behavioral improvement in at 6 months

Prior to the onset of COVID and the resultant cancellation of live classroom sessions, the SDQ was filled out by the CLC teaching staff. In total, there were 21 teacher reports that spanned enrollment to the 6- month time point (controls, *n* = 11; MCEP, *n* = 10).

**Figure 6:**
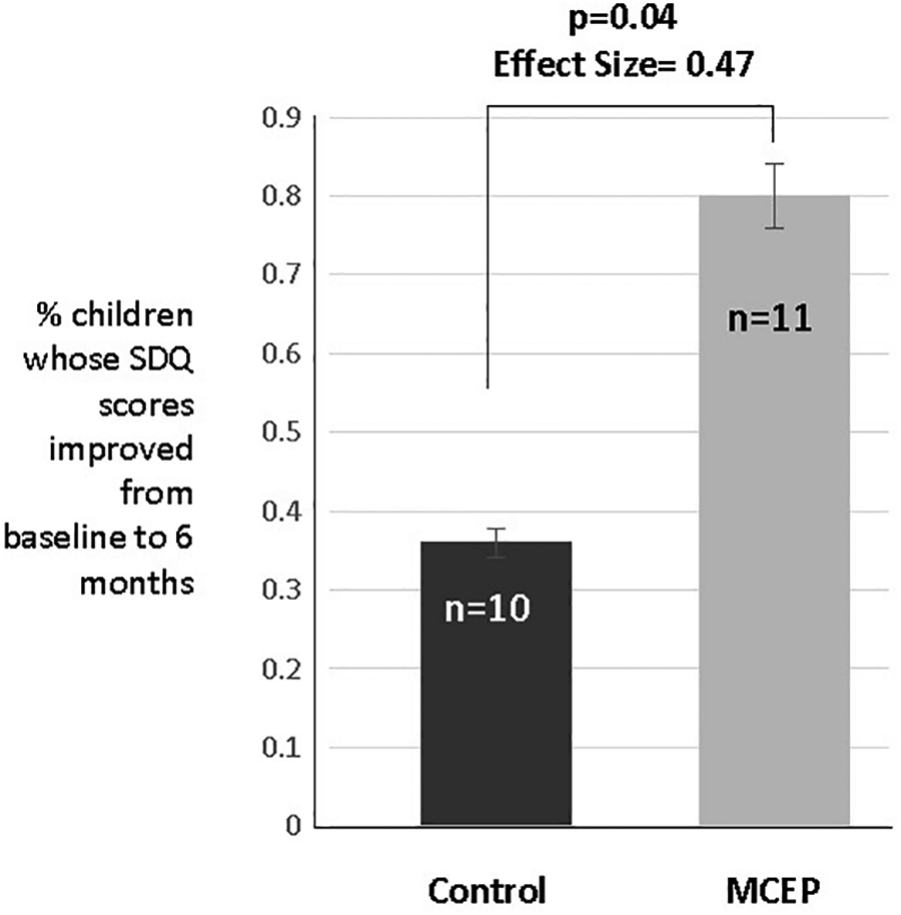
Teacher rated SDQ. According to the teacher, the percentage of control children which showed a decline in total difficulties was 36% (4 of 11) vs. 80% in MCEP (8 of 10); *X*^2^ = 4.07, *p* = 0.04, effect size (Fisher's *Z* = 0.47). SDQ, strength and difficulties questionnaire; MCEP, Mother-child emotional preparation.

At the time of enrollment (baseline) the total SDQ scores for emotional behavioral disorders did not differ between controls (mean = 7.6, SD = 5.0) and MCEP (mean = 8.5, SD = 4.6), *t* = 0.46, *p* = 0.65). The change (0–6 months) in SDQ Total Difficulties Score (Total Score = Emotional Scale + Conduct Scale + Hyperactivity Scale + Peer Problem Scale) approached statistical significance (*t* = 1.89, *p* = 0.076), with MCEP children showing greater declines in problems from baseline to six-months (control mean = + 0.82, SD = 5.96; MCEP mean = −3.30, SD = 3.92) with an effect size (Cohen's *d*) of 0.76. The percentage of control children which showed a decline in Total Difficulties was 36% in controls (4 of 11) vs. 80% in MCEP (8 of 10; *X*^2^ = 4.07, *p* = 0.044, effect size (Fisher's *Z* = 0.47) (see Figure 6).

For 20 of these 21 teacher reports, we also had parent reports. Concordant with the teacher reports, mother SDQ reports indicated 40% (4 of 10) of the control and 80% (8 of 10) of the MCEP children showed reductions in total EBD scores from enrollment to the 6-month time point. For baseline (enrollment) SDQ EBD total scores, there was a good correlation between parent (mother) and teacher scores. Pre-COVID cohort, *N* = 29 (control and MCEP combined), *r* = 0.53, *p* = 0.003. This indicates a general agreement between mothers and teachers on how children were behaving. During COVID teachers reports could not reflect school behavior as children were remote.

### Maternal depressive symptoms (CES-D)

We analyzed CES-D scores without controlling for various covariates and found no significant reduction in mother- reported depressive symptoms in either group. However, we considered whether there might be factors that could impact CES-D scores that need to be taken into account. Accordingly, we re-ran ANOVAs on 6-month scores for several possible covariates; child sex, maternal age, pre/post COVID, child age at enrollment, ACES scores. Of these covariates, two were found to be significant correlates of CES-D scores at 6 months, child age and the ACES score. When these two variables were included in the model, the effects of MCEP were found to be significant [*F*(1,63) = 5.34, *p* = 0.024]. To better understand how these covariates impacted the effects of MCEP on maternal depressive systems we ran post-hoc tests in which subjects were stratified by age and in which extreme values of ACES scores (>5, *n* = 6) were excluded.

We found that mothers of children over four had higher CESD scores. The first of these analyses showed that the effects of MCEP on reducing CES-D scores were significant for mothers with children <4 years of age (control *n* = 20, mean = 12.1 ± 10.0SD; MCEP *n* = 23, mean = 6.5 ± 6.6SD, *t* = 2.19, *p* = 0.035, ES = 0.60). For mothers with children of age there was no significant effect of MCEP and CES-D scores were lower than for the younger cohort (control *n* = 13, mean = 4.9 ± 5.6SD; MCEP *n* = 11, mean = 4.4 ± 6.5SD, *t* = 2.19, *p* = 0.035, ES = 0.60). Thus, MCEP was found to reduce maternal symptoms but not in mothers of older children whose CES-D scores were much lower at baseline.

In the second analysis we excluded the 6 mothers who reported high levels of childhood trauma 3 control (mean CES-D = 20.0) and 3 MCEP (mean CES-D = 17.3). After removing these 6 subjects, the effects of MCEP were significant (control *n* = 30, mean = 8.2 ± 7.8SD; MCEP *n* = 31, mean = 4.7 ± 4.2SD, *t* = 2.17, *p* = 0.035, ES = 0.59). These analyses suggest that the depressive symptoms of women with high levels of childhood trauma were resistant to the positive effects of MCEP. However, most women showed decreases in depressive symptoms 6 months after entering the MCEP program.

### School staff survey showed widespread support for continuing the MCEP program

**Figure 7:**
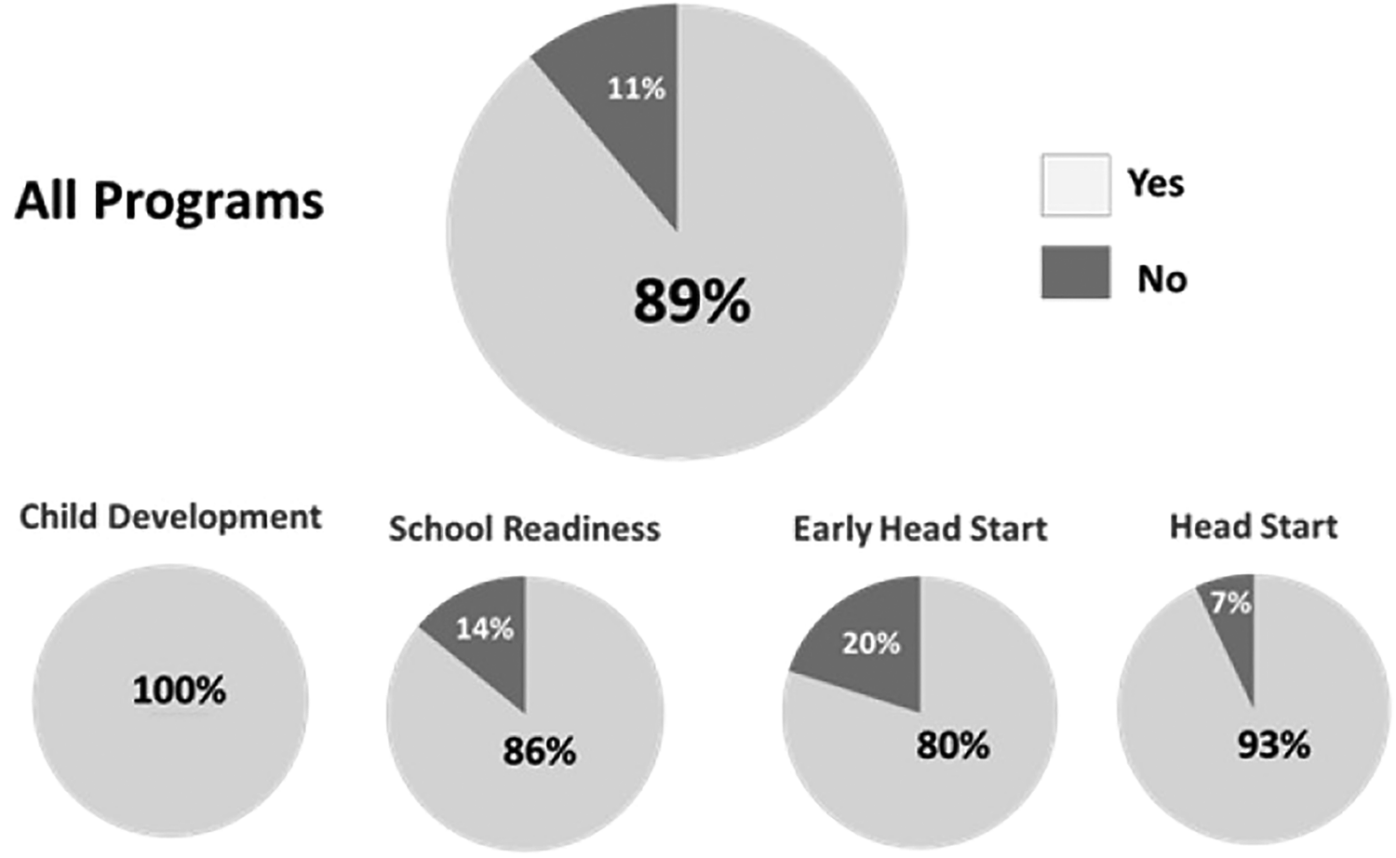
Results of a survey conducted among CLC preschool education staff following the completion of the randomized controlled trial. Question: “Should the MCEP program be integrated into the standard curriculum?” CLC, Children's Learning Centers of Fairfield County; MCEP, mother-child emotional preparation.

During the second half of the MCEP study, CLC staff were asked if they thought this program should be continued. A total of 72 teachers and staff responded and 89% supported continuation of MCEP and its integration into CLC (see Figure 7).

## Discussion

The results of this RCT conducted within a community-based preschool education curriculum show that the group MCEP intervention model was successfully implemented and resulted in significant positive changes in emotional connection and in both parent and teacher-reported child behavior at home and in the classroom. We found that MCEP increased the percentage of emotionally connected dyads 6 months after enrollment in the study, as measured by the Welch Emotional Connection Screen (WECS) assessment tool. The emotional connection construct describes a shared “behavioral state”, which is measurable via the WECS. As stated in the methods section, the emotional connection construct was validated in a prematurely born sample at 4 months of age. Our findings presented here validate the emotional connection construct in a preschool-aged child population.

We found that MCEP dyads not connected at the time of enrollment were five times more likely to become emotionally connected by the 6-month follow-up. In addition, dyads in the intervention group showed ∼50% reduction in behavioral problems as measured on the SYWC assessment measure. There were also less symptomatic emotional behaviors (SDQ), fewer symptoms related to autism (ATEC), improved socio-emotional function (ASQ), and greater cognitive flexibility (Brief-P).

We referred to this preschool intervention as Mother-child Emotional Preparation- (MCEP) to distinguish it from Family Nurture Intervention in neonatal intensive care unit model (FNI-NICU), which Welch and team designed for mothers and prematurely born infants. Both FNI-NICU and MCEP are based on the same calming cycle theoretical framework, which is not age-specific (26).

The FNI-NICU model was designed to overcome mother and infant autonomic stress and dysregulation after premature birth. The intervention was adapted from clinical practices developed by Welch in the 1970's (38). RCTs of FNI-NICU at Morgan Stanley Children's Hospital of New York (ClinicalTrials.gov identifier NCT01439269) showed that intervention babies had significantly accelerated brain development and autonomic regulation by term age (39–41), and that results at term age correlated with significantly improved neurodevelopment, social-relatedness, and attention at the 18-month follow-up (42). In addition, mothers of intervention babies showed improved quality of maternal care (42) and improved maternal anxiety and depressive symptoms at 4-months (23). Both FNI mothers and children had better physiological regulation at 5 years (43). The results show that an intervention aimed at enhancing mother-infant emotional connection can improve biobehavioral functioning and developmental trajectories of preterm infants.

Studies have demonstrated that child behavior and physiology are mediated by family and community relationships (44, 45). These relationships influence the child's socioemotional and physiological wellbeing. The mother-child relationship serves as the primary biological and psychological stress-modulating relationships (47–48). Thus, a stressful mother-child relationship can impair the child's socioemotional and neurological development, and stress responsivity (49, 50). It is crucial to repair a suboptimal mother-child relationship as early as possible during development to avoid chronic activation of their stress response systems (51).

Many current therapies focus on behavior and stress responses of the dysregulated child apart from the mother (52) Such therapies aim to help the child self-manage emotions and emotion-related motivational and physiological states in order to change the child's behavior and temperament (53). At the core of temperamental regulation is a construct of effortful control, defined as “the efficiency of executive attention—including the ability to inhibit a dominant response and/or to activate a subdominant response—to plan, and to detect errors” (54).

As noted in the introduction, there are many tested interventions for preschool children. However, none of these has leveraged the power of engaging both mother and child together in developing emotional connection and co- regulation (vs. self-regulation) in a school-based program and none have shown the breadth of improvements demonstrated using MCEP. MCEP is novel in active engagement of both mother and child in early childhood relationship-based intervention paving the way for sustained effects on child development and relational health (23, 26, 28, 39, 41–43). Results of this MCEP trial add to evidence that calming cycle theory and mother-infant emotional connection set the foundation for optimal child emotional and behavioral development.

In many interventions in this age group in similar settings, the child is treated separately from the parent. Teachers often serve as parental stand-ins and focus on self-regulatory activities (55–58). These interventions aim to enhance teacher-student relationships and improve child classroom behaviors by helping the child to better self-regulate emotions.

Other programs, such as Nurse Family Partnership target the foundational mother-child relationship in the home to support better socioemotional behavior in the classroom (59). A limited number of Head Start studies of programs that allow parents to become more directly engaged in additional parent-child activities, such as parent-child play, reading bedtime routines, reading daily, and reading frequency, have shown an overall positive effect on classroom behavior (60).

CLC currently employs the RULER (61) school-based teaching program and strategy within its standard curriculum to improve adverse child classroom behavior. RULER is designed to modify the quality of classroom social interactions, so that the social climate becomes more supportive, empowering, and engaging. This is accomplished by teaching children emotion regulation by solidifying their emotion identification skills, and integrating skill-building lessons and tools so that teachers and students develop their emotional literacy. The teaching-focused RULER model proved somewhat effective in the classroom. However, CLC staff reported the program was not keeping up with the growing numbers and severity of dysregulated child behavior in the classroom.

In this group model, mothers and children engaged in calming sessions at school and continued in the home prior to encountering the daily pre-school socioemotional classroom experience. The intervention is based on calming cycle theory (26, 27), which posits that child socioemotional behavior is autonomic state- dependent (as opposed to psychological trait-dependent). Symptomatic anti-socioemotional behavior in the home and at school is the result of autonomic state dysregulation. MCEP changes anti-socioemotional behavior to pro- socioemotional behavior through regular parent-child calming sessions that reinstate adaptive parent-child co-regulation of the child's and mother's autonomic states. Once established, the co-regulation may extend to school teachers and staff and pro-socioemotional behaviors emerge. Note the high percentage of CLC teachers (89%) (Figure 7) who want MCEP to be included in the standard curriculum MCEP involves fostering close, authentic and reciprocal emotionally calming interactions between mother and child during group sessions. MCEP targets mutual mother-child emotional connection as a powerful mediator of the dyad's ability to cope with stress (26, 27).

MCEP is novel in its engagement of both mother and child in a full range of emotional expression during mutual sensory calming sessions. Our hypothesis is that empathy is evoked when the mother (or other person) expresses the full range of their deep feelings. When a child responds to the mother's expression of emotion and shows signs of empathy to the mother, a temporary break in connection begins to repair. Repair needs to be mutual and on-going to be effective for both members of the pair. Both the mother and child learn that repair can be achieved through emotional communication. The connected child is thereafter able to connect and co-regulate with teachers and others once the pattern is established with the mother and family members. CLC leadership has reported that the children who were in the MCEP program changed the classroom environment with their positive affect and better regulated behavior so much so that teachers were freer to address problems such as learning difficulties of the non- symptomatic children for whom teachers did not have the band-width to address prior to MCEP.

### Limitations

Subjects for this study were a non-probability convenience sample of mainly low socioeconomic status, which could limit result generalizability. Their lack of access to behavioral, emotional and developmental care options may have limited the effect sizes of MCEP measures. We conducted several secondary and ancillary analyses. As no penalty for multiple assessments were applied to these tests, our results should be considered preliminary. However, one of the two prospectively declared outcome tests for emotional connection (time-point 2), was found to be significant at the 0.015 level. COVID interrupted and ended many of the follow-ups. Nonetheless, it is important to note that effect sizes on all the outcomes were substantial and of potentially great importance to the well-being of the child, mother and their relational health.

## Conclusion

The calming sessions employed in MCEP engage mothers and children in face-to-face interaction in a mutually calming way that achieves emotional connection without the need for expensive curriculum or toys/stimuli. The simplicity of MCEP underlies both the scalability and the efficacy of the program. The skills acquired by the pair generalize to home readily, as minimal time is required to reconnect emotionally and mutually calm with each other each day.

As well, MCEP techniques are feasible and actionable, because expenses are minimal and techniques may be applied in multiple settings or at any time point. Improvements in mother-child emotional connection and in child behavior were significant, with large effect sizes. The fact that these effects were independent of maternal ACES is highly meaningful in this challenged preschool population. MCEP is easily scalable to other community preschools as well as to kindergartens because it could be executed by the schools’ own childhood professional staff trained in the MCEP program.

### Future directions

While the MCEP program also showed improvement in maternal depressive symptoms, this effect was moderated by a maternal history of childhood adversity. Given the small number of participants reporting high ACES in this study, it is imperative to do future research with a larger sample size. It would be especially fruitful to recruit women who have a history of childhood adversity, who may be struggling with childhood behavioral problems at home. Future studies will include other important variables, such as the efficacy of a Father-child emotional preparation program. Remote schooling during COVID prompted the development of a virtual model of MCEP. We are currently conducting feasibility trials of the virtual MCEP model to test its efficacy and effectiveness. While challenging initially, preliminary results suggest that a remote, internet-based platform speeds dissemination and makes MCEP more accessible, not just to mother and child but to the whole family at home. Moreover, a virtual model may prove generalizable to diverse populations without compromising efficacy and effectiveness

## Data Availability

The original contributions presented in the study are included in the article/**Supplementary Material**, further inquiries can be directed to the corresponding author.

## References

[1] Kupersmidt JB Bryant D Willoughby MT. Prevalence of aggressive behaviors among preschoolers in Head Start and community care programs. Behavioral Disorders. (2000) 26(1):42–52.

[2] Zaim N Harrison J. Pre-school mental health disorders: a review. Int Rev Psychiatry. (2020) 32(3):189–201. 10.1080/09540261.2019.1692793 31814465

[3] Barry CT Malkin ML. The assessment of antisocial behavior in children and adolescents. New York: Oxford Academic (2012).

[4] Rutter M Giller H Hagell A. Antisocial behavior by young people. Camridge: Cambridge University Press (1998).

[5] Foster EM Jones DE. The high costs of aggression: public expenditures resulting from conduct disorder. Am J Public Health. (2005) 95(10):1767–72. 10.2105/AJPH.2004.061424 16131639 PMC1449434

[6] Romeo R Knapp M Scott S. Economic cost of severe antisocial behaviour in children--and who pays it. Br J Psychiatry. (2006) 188:547–53. 10.1192/bjp.bp.104.007625 16738345

[7] Scott S Knapp M Henderson J Maughan B. Financial cost of social exclusion: follow up study of antisocial children into adulthood. BMJ. (2001) 323(7306):191. 10.1136/bmj.323.7306.191 11473907 PMC35269

[8] Adoptaclassroom.org. State of teaching statistics. (2022).

[9] Tourjman V Louis-Nascan G Ahmed G DuBow A Cote H Daly N Psychosocial interventions for attention deficit/hyperactivity disorder: a systematic review and meta-analysis by the CADDRA Guidelines Work GROUP. Brain Sci. (2022) 12(8). 10.3390/brainsci12081023 36009086 PMC9406006

[10] Ooi J Dodd HF Meiser-Stedman R Hudson JL Bridges J Pass L. The efficacy of interventions for behaviourally inhibited preschool-aged children: a meta-analysis. J Anxiety Disord. (2022) 88:102559. 10.1016/j.janxdis.2022.102559 35366584

[11] James AC Reardon T Soler A James G Creswell C. Cognitive behavioural therapy for anxiety disorders in children and adolescents. Cochrane Database Syst Rev. (2020) 11(11):CD013162. 10.1002/14651858.CD013162.pub2 33196111 PMC8092480

[12] Caldwell DM Davies SR Hetrick SE Palmer JC Caro P Lopez-Lopez JA School-based interventions to prevent anxiety and depression in children and young people: a systematic review and network meta-analysis. Lancet Psychiatry. (2019) 6(12):1011–20. 10.1016/S2215-0366(19)30403-1 31734106 PMC7029281

[13] Korest R Carlson JS. A meta-analysis of the current state of evidence of the incredible years teacher-classroom management program. Children (Basel). (2021) 9(1). 10.3390/children9010024 35053649 PMC8774151

[14] Colalillo S Johnston C. Parenting cognition and affective outcomes following parent management training: a systematic review. Clin Child Fam Psychol Rev. (2016) 19(3):216–35. 10.1007/s10567-016-0208-z 27389605

[15] Valero-Aguayo L Rodriguez-Bocanegra M Ferro-Garcia R Ascanio-Velasco L. Meta-analysis of the efficacy and effectiveness of parent child interaction therapy (PCIT) for child behaviour problems. Psicothema. (2021) 33(4):544–55. 10.7334/psicothema2021.70 34668468

[16] Donohue MR Yin J Quinones-Camacho L Hennefield L Tillman R Gilbert K Children’s maternal representations moderate the efficacy of parent-child interaction therapy-emotion development (PCIT-ED) treatment for preschool depression. Res Child Adolesc Psychopathol. (2022) 50(9):1233–46. 10.1007/s10802-022-00897-2 35133556 PMC9808883

[17] Bayer JK Rapee RM Hiscock H Ukoumunne OC Mihalopoulos C Clifford S The Cool Little Kids randomised controlled trial: population-level early prevention for anxiety disorders. BMC Public Health. (2011) 11:11. 10.1186/1471-2458-11-11 21208451 PMC3027133

[18] Ray C Kaukonen R Lehto E Vepsalainen H Sajaniemi N Erkkola M Roos E. Development of the DAGIS intervention study: a preschool-based family-involving study promoting preschoolers' energy balance-related behaviours and self-regulation skills. BMC Public Health. (2019) 19(1):1670. 10.1186/s12889-019-7864-0 31830926 PMC6909522

[19] Kochanska G. Mutually responsive orientation between mothers and their young children: implications for early socialization. Child Dev. (1997) 68(1):94–112. 9084128

[20] Joo YS Magnuson K Duncan GJ Schindler HS Yoshikawa H Ziol-Guest KM. What works in early childhood education programs?: a meta–analysis of preschool enhancement programs. Early Educ Dev. (2019) 31(1):1–26. 10.1080/10409289.2019.1624146

[21] Barlow J Bergman H Kornor H Wei Y Bennett C. Group-based parent training programmes for improving emotional and behavioural adjustment in young children. Cochrane Database Syst Rev. (2016) 2016(8):CD003680. 10.1002/14651858.CD003680.pub3 27478983 PMC6797064

[22] Jeong J Pitchik HO Fink G. Short-term, medium-term and long-term effects of early parenting interventions in low- and middle-income countries: a systematic review. BMJ Glob Health. (2021) 6(3). 10.1136/bmjgh-2020-004067 33674266 PMC7938974

[23] Welch MG Halperin MS Austin J Stark RI Hofer MA Hane AA Depression and anxiety symptoms of mothers of preterm infants are decreased at 4 months corrected age with Family Nurture Intervention in the NICU. Arch Womens Ment Health. (2016) 19(1):51–61. 10.1007/s00737-015-0502-7 25724391

[24] Schulz KF Altman DG Moher D, Group C. CONSORT 2010 statement: updated guidelines for reporting parallel group randomised trials. BMJ. (2010) 340:c332. 10.1136/bmj.c332 20332509 PMC2844940

[25] Children's Learning Centers of Fairfield County. Children’s learning centers of fairfield county parent manual. (2016). Available at: http://www.clcfc.org/wp-content/uploads/2017/05/Parent-Manual-Version-1.0-English-Final-11.22.2016.pdf

[26] Welch MG. Calming cycle theory: the role of visceral/autonomic learning in early mother and infant/child behaviour and development. Acta Paediatr. (2016) 105(11):1266–74. 10.1111/apa.13547 27536908

[27] Welch MG Ludwig RJ. Calming cycle theory and the co-regulation of oxytocin. Psychodyn Psychiatry. (2017) 45(4):519–40. 10.1521/pdps.2017.45.4.519 29244620

[28] Hane AA LaCoursiere JN Mitsuyama M Wieman S Ludwig RJ Kwon KY The welch emotional connection screen: validation of a brief mother-infant relational health screen. Acta Paediatr. (2019) 108(4):615–25. 10.1111/apa.14483 29959878

[29] Frosch CA Fagan MA Lopez MA Middlemiss W Chang M Hane AA Validation study showed that ratings on the Welch Emotional Connection Screen at infant age six months are associated with child behavioural problems at age three years. Acta Paediatr. (2019) 108(5):889–95. 10.1111/apa.14731 30702768

[30] Ludwig RJ Welch MG. Wired to connect: the autonomic socioemotional reflex arc. Front Psychol. (2022) 13:841207. 10.3389/fpsyg.2022.841207 35814106 PMC9268160

[31] Radloff LS. The CES-D scale: a self-report depression scale for research in the general population. Appl Psychol Meas. (1977) 1(3):385–401.

[32] Perrin EC Sheldrick C Visco Z Mattern K. The survey of well-being of young children (SWYC) user’s manual. (2016). Available at: https://www.tuftschildrenshospital.org/-/media/Brochures/Floating-Hospital/SWYC/SWYC-Manual-v101-Web-Format-33016.ashx?la=en&hash=E0C2802F003ED312E9D5268374C540A112151FB3

[33] Rimland B Edelson M. Autism treatment evaluation checklist San Diego. (1999). Available at: https://www.autismeval.com/ari-atec/report1.html

[34] Goodman R. The strengths and difficulties questionnaire: a research note. J Child Psychol Psychiatry. (1997) 38(5):581–86. 10.1111/j.1469-7610.1997.tb01545.x 9255702

[35] Bricker D Squires J Kaminski R Mounts L. The validity, reliability, and cost of a parent-completed questionnaire system to evaluate at-risk infants. J Pediatr Psychol. (1988) 13(1):55–68. 10.1093/jpepsy/13.1.55 2455033

[36] Squires J Bricker D Potter L. Revision of a parent-completed development screening tool: ages and stages questionnaires. J Pediatr Psychol. (1997) 22(3):313–28. 10.1093/jpepsy/22.3.313 9212550

[37] Gioia GA Isquith PK Retzlaff PD Espy KA. Confirmatory factor analysis of the Behavior Rating Inventory of Executive Function (BRIEF) in a clinical sample. Child Neuropsychol. (2002) 8(4):249–57. 10.1076/chin.8.4.249.13513 12759822

[38] Welch MG Chaput P. Mother-child holding therapy and autism. Pennsylvania medicine. (1988) 91(10):33–8. 3226740

[39] Isler JR Stark RI Grieve PG Welch MG Myers MM. Integrated information in the EEG of preterm infants increases with family nurture intervention, age, and conscious state. PLoS One. (2018) 13(10):e0206237. 10.1371/journal.pone.0206237 30356312 PMC6200276

[40] Porges SW Davila MI Lewis GF Kolacz J Okonmah-Obazee S Hane AA. Autonomic regulation of preterm infants is enhanced by Family Nurture Intervention. Dev Psychobiol, (2019) 61(6):942–52. 10.1002/dev.21841 30868570

[41] Welch MG Myers MM Grieve PG Isler JR Fifer WP Sahni R Electroencephalographic activity of preterm infants is increased by Family Nurture Intervention: a randomized controlled trial in the NICU. Clin Neurophysiol. (2014) 125(4):675–84. 10.1016/j.clinph.2013.08.021 24140072

[42] Welch MG Firestein MR Austin J Hane AA Stark RI Hofer MA Family nurture intervention in the neonatal intensive care unit improves social-relatedness, attention, and neurodevelopment of preterm infants at 18 months in a randomized controlled trial. J Child Psychol Psychiatry. (2015) 56(11):1202–11. 10.1111/jcpp.12405 25763525

[43] Welch MG Barone JL Porges SW Hane AA Kwon KY Ludwig RJ Family nurture intervention in the NICU increases autonomic regulation in mothers and children at 4-5 years of age: follow-up results from a randomized controlled trial. PLoS One. (2020) 15(8):e0236930. 10.1371/journal.pone.0236930 32750063 PMC7402490

[44] Gong W Rolls ET Du J Feng J Cheng W. Brain structure is linked to the association between family environment and behavioral problems in children in the ABCD study. Nat Commun. (2021) 12(1):3769. 10.1038/s41467-021-23994-0 34145259 PMC8213719

[45] Zhao H Cheng T Zhai Y Long Y Wang Z Lu C. How mother-child interactions are associated with a child’s compliance. Cereb Cortex. (2021) 31(9):4398–410. 10.1093/cercor/bhab094 33895811

[46] Francis DD Meaney MJ. Maternal care and the development of stress responses. Curr Opin Neurobiol. (1999) 9(1):128–34. 10.1016/s0959-4388(99)80016-6 10072372

[47] NICHD Early Child Care Research Network. Affect dysregulation in the mother-child relationship in the toddler years: antecedents and consequences. Dev Psychopathol. (2004) 16(1):43–68. 10.1017/s0954579404044402 15115064

[48] Rincon-Cortes M Sullivan RM. Early life trauma and attachment: immediate and enduring effects on neurobehavioral and stress axis development. Front Endocrinol (Lausanne). (2014) 5:33. 10.3389/fendo.2014.00033 24711804 PMC3968754

[49] Hane AA Fox NA. Early caregiving and human biobehavioral development: a comparative physiology approach. Curr Opin Behav Sci. (2016) 7:82–90. 10.1016/j.cobeha.2015.12.002 26753173 PMC4703360

[50] Tang AC Reeb-Sutherland BC Romeo RD McEwen BS. On the causes of early life experience effects: evaluating the role of mom. Front Neuroendocrinol. (2014) 35(2):245–51. 10.1016/j.yfrne.2013.11.002 24246856

[51] Slopen N Kubzansky LD McLaughlin KA Koenen KC. Childhood adversity and inflammatory processes in youth: a prospective study. Psychoneuroendocrinology. (2013) 38(2):188–200. 10.1016/j.psyneuen.2012.05.013 22727478 PMC3632283

[52] Moltrecht B Deighton J Patalay P Edbrooke-Childs J. Effectiveness of current psychological interventions to improve emotion regulation in youth: a meta-analysis. Eur Child Adolesc Psychiatry. (2021) 30(6):829–48. 10.1007/s00787-020-01498-4 32108914 PMC8140974

[53] Eisenberg N Hofer C Vaughan J. Effortful control and its socioemotional consequences. New York: Guilford Press (2007).

[54] Rothbart MK Bates JE. Temperament. New York: Wiley (2006).

[55] Bellone KM Dufrene BA Tingstrom DH Olmi DJ Barry C. Relative efficacy of behavioral interventions in preschool children attending head start. J Behav Educ. (2014) 23(3):378–400. 10.1007/s10864-014-9196-6

[56] Gerholm T Kallioinen P Toner S Frankenberg S Kjallander S Palmer A A randomized controlled trial to examine the effect of two teaching methods on preschool children’s language and communication, executive functions, socioemotional comprehension, and early math skills. BMC Psychol. (2019) 7(1):59. 10.1186/s40359-019-0325-9 31488204 PMC6729003

[57] McLaren EM Nelson CM. Using functional behavior assessment to develop behavior interventions for students in head start. J Posit Behav Interv. (2009) 11(1):3–21. 10.1177/1098300708318960

[58] Zhai F Raver CC Li-Grining C. Classroom-based interventions and teachers' perceived job stressors and confidence: evidence from a randomized trial in head start settings. Early Child Res Q. (2011) 26(7):442–52. 10.1016/j.ecresq.2011.03.003 21927538 PMC3172132

[59] Williams VN McManus BM Brooks-Russell A Yost E Allison MA Olds DL A qualitative study of effective collaboration among nurse home visitors, healthcare providers and community support services in the United States. Health Soc Care Community. (2022) 30(5):1881–93. 10.1111/hsc.13567 34543476

[60] Alotta J. The effects of head start on parenting: A systematic literature review. Albany, New York: SUNY (2020). Available at: https://scholarsarchive.library.albany.edu/honorscollege_pad/12

[61] Brackett MA Rivers SE Reyes MR Savoley P. Enhancing academic performance and social and emotional competence with the RULER feeling words curriculum. J Individ Differ. (2012) 22:218–24. 10.1016/j.lindif.2010.10.002

